# Asthma and Food Allergy in Children: Is There a Connection or Interaction?

**DOI:** 10.3389/fped.2016.00034

**Published:** 2016-04-05

**Authors:** Carlo Caffarelli, Marilena Garrubba, Chiara Greco, Carla Mastrorilli, Carlotta Povesi Dascola

**Affiliations:** ^1^Clinica Pediatrica, Department of Clinica and Experimental Medicine, Azienda Ospedaliero-Universitaria di Parma, University of Parma, Parma, Italy

**Keywords:** anaphylaxis, asthma, children, food allergy, IgE, skin prick test, wheezing

## Abstract

This review explores the relationship between food allergy and asthma. They can share the same risk factors, such as parental allergy, atopic eczema, and allergen sensitization, and they often coincide in the same child. Coexistence may negatively influence the severity of both conditions. However, it remains to be determined whether food allergy may directly affect asthma control. An early food sensitization in the first year of life can predict the onset of asthma. Furthermore, asthmatic symptoms could rarely be caused by ingestion or inhalation of the offending food. Asthma caused by food allergy is severe and may be associated with anaphylactic symptoms. Therefore, an accurate identification of the offending foods is necessary in order to avoid exposure. Patients should be instructed to treat asthmatic symptoms quickly and to use self-injectable epinephrine.

## Introduction

The management of children with food allergy and asthma is a growing concern. Prevalence of both conditions is high even if variable among populations and among heterogeneous studies. There is evidence that the prevalence has increased over recent decades. Asthma affects about 9% of children in the world. Its frequency fluctuates from 1 to 30% among countries, being higher in Western countries (1). The self-reported prevalence of food allergy varies from 3 to 35% in childhood (2). However challenge-proved food allergy provides lower estimates, range 1–10.8% (3). Asthma and food allergy are in a close relationship. They can share the same risk factors, such as parental allergy, atopic eczema, and allergen sensitization. Population studies have shown that an early food sensitization (4) or food allergy (5) in the first year of life precedes the development of asthma (4) and atopic diseases, such as atopic dermatitis, allergic rhinitis, and allergic conjunctivitis, the so-called atopic march, as well as allergy to other foods (food allergen march) at school age (5).

Asthma and food allergy may coexist, and this adversely influences their course. Furthermore, asthma attack can be elicited by food allergens in sensitized children. Primary food sensitization may occur through the intestinal route because of the immaturity of the intestinal barrier and of the immune system in infants (6). However, other routes of sensitization have been proposed. In children with atopic eczema, topical application of ointments containing peanut oil has been associated with a significant increase of peanut allergy frequency (7). In mice (8), it has been shown that early feeding with food allergens induces tolerance, while the exposure of inflamed skin to food induces sensitization that leads to an allergic intestinal reaction when the food is ingested (“double exposure” hypothesis) (9). Accordingly, peanut consumption within the family is closely related to the amount of peanut protein measurable on cradle and toys (10), and it is a risk factor for the onset of peanut allergy in childhood (11). The onset of peanut allergy in high-risk children is prevented by a not delayed introduction of peanuts from 4 months of age to 11 months (12, 13). Foods can act as aeroallergens, and some patients may be sensitized by inhaled food particles. In agreement, it has been shown that inhaled peanut and shrimp elicit a clinical hypersensitivity reaction in children who have never previously ingested these foods (13). The following sections review the most relevant publication from the perspective of the clinician on the manner, in which food allergy may regulate the development of asthma and on the direct role of food allergy on asthma. Relevant papers on the topic were either familiar to the authors or they were selected by carrying out a search using the PubMed database.

## Comorbidity of Asthma and Food Allergy

Consistent evidence from observational studies associates asthma with food allergy. The frequency of food sensitization in asthmatic children is higher than expected in a general population (14, 15). Asthmatic patients show specific IgE antibodies >0.35 kU/L to at least one of the six more allergenic foods in the US (egg, milk, soy, peanut, wheat, and fish) in 45% of cases (14). Liu et al. (15) reported that the risk for being sensitized to milk, egg, peanut, shrimp, or multiple foods is higher in asthmatic patients. Schroeder at al. (16) showed that symptomatic sensitization to foods was associated with asthma both in children <6 years of age and >6 years of age, especially in children with allergy to more than two foods or severe food allergies. On the other hand, children with anaphylactic reactions to foods often present asthma. Schroeder at al. (16) showed that clinical reactions to foods suggestive of anaphylaxis were associated with diagnosis of asthma.

Several findings suggest that coexistence of asthma and food allergy increases the severity of the disorders. The risk of asthma morbidity, including daytime symptoms, hospital admissions, and lower percent predicted forced expiratory volume in 1-s (FEV1) values, was higher in children with food allergy. This risk is even greater when food allergy was multiple or severe (17). Moreover, IgE sensitization to foods is linked to more severe asthma. Asthmatics with at least one food sensitization are more likely to be admitted to hospital, to use corticosteroids (14), and to have emergency department visits for asthma in comparison with those not sensitized to any food (15). An increase in food sensitization and food allergy is indicative of asthma persistence and severe asthma exacerbations (15). A strong relationship between the number of offending foods and asthma attacks has been reported (16). Arabkhazaeli et al. (18) analyzed 703 children from the PACMAN cohort who regularly used asthma medication. Children with coexisting allergic conditions (hay fever, food allergy, and eczema) were at greater risk of severe asthma. They also found a significantly higher use of oral corticosteroids in subjects with food allergy compared with the non-food allergy population (9.6 vs. 4.3%). Vogel et al. (19) compared 72 children with severe asthma admitted to pediatric intensive care unit (PICU) with patients with mild asthma. They showed that asthmatics admitted to the PICU were younger and significantly more likely to report food allergy. Nonetheless, most of previous reports on distinguishing features of children with severe asthma have not studied food allergy as a marker of severity (20–22). Recently, Just et al. (23) identified a novel phenotype of severe asthma in children characterized by more common sensitizations to inhaled allergens and food allergens, higher circulant eosinophils and basophils, lack of effect of high doses of inhaled corticosteroid, and more frequent hospital admissions for acute attacks. Conversely, Konradsen (24) did not find that food allergy was more common in children with problematic severe asthma in comparison with those with controlled persistent asthma.

Finally, it has been found that asthma is a risk factor for severe or fatal anaphylactic reactions to foods. A key study showed that asthma is the main cause of death in children with anaphylaxis to foods (25).

Another issue is whether in children presenting both asthma and food allergy, asthma management should be more aggressive. Recent studies have shown higher fractional exhaled nitric oxide (FeNO) and sputum eosinophil percentage in asthmatic children with food allergy compared with asthmatic children without allergies (26). Moreover, adults sensitized both to pollens and food of plant origin have increased risk for asthma and elevated levels of FeNO compared with subjects sensitized only to pollens (27). In asthmatics, an increase in FeNO, airway responsiveness, and circulating eosinophils is associated with positive IgE to wheat, peanut, soy, shrimp, egg, milk, and fish (28). Such data highlight that asthmatics with food sensitization have higher degree of eosinophilic inflammation in the airways. Therefore, they should be more closely monitored and perhaps need more treatment (29).

## Food Allergy and Prediction of Asthma Development

A collective body of evidence suggests that there is a progression from atopic eczema and food allergy, that have the highest incidence in the first 2 years of life, to wheezing (30), asthma (4, 31), and sensitization to inhalant allergens at school age.

Some studies have revealed that children with atopic eczema who are sensitized to egg were at higher risk for later development of asthma (32). An English working group found that in an unselected children cohort at 4 years old, egg allergy based on history and positive skin tests was associated with respiratory symptoms, rhinitis, and asthma, especially when coexisting with eczema (33). In addition, it was observed that positive egg-specific IgE antibodies in eczematous infants were associated with an increased risk for developing IgE for inhalant during the next 3 years (31). The risk for asthma is greater in eczematous infants with sensitization to a panel of food allergens and filaggrin mutation (34).

The question also arises whether food allergy is a predictor of the onset of asthma independently from atopic eczema. It has been observed that IgE sensitization to foods or aeroallergens anticipate the appearance of wheezing at age 7, regardless of eczema in first years of life (4). Moreover, it has been showed that early sensitization to egg may predict asthma occurrence in the general population (33). Rhodes et al. (35) followed up 63 patients with familiar history of atopic disease from birth to 22 years of age. Twenty patients had positive skin prick test results for cow’s milk and/or egg in the first year of life. Food sensitization was associated with later asthma (4). Infants with allergy to egg, cow’s milk, and wheat are at risk for development of asthma (5). The risk did not differ significantly between those who developed and did not develop tolerance to egg, cow’s milk, and wheat by 3 years old (5). In children with wheezing, sensitization to egg white (14, 36) or wheat (21) was a risk factor for developing asthma.

Schroeder at al. (16) studied a family-based food allergy cohort of 567 children. They found that patients with allergy to cow’s milk, egg, peanut, and nuts developed more frequently and earlier asthma than children without food allergy. Priftis et al. (37) conducted a case–control study in 69 children with allergy to egg and/or fish in the first 3 years of life. They reported that children with food allergy in infancy were at increased risk for wheezing and bronchial hyperreactivity (BHR) during school age. Asthma symptoms were reported more frequently in the study group than in the control group, which enrolled children sensitized and non-sensitized to aeroallergens. Children of the study group showed a significantly increased frequency of positive response to methacholine bronchial challenge compared to the control group. The importance of food allergy as risk factor for asthma onset in young children has been confirmed by the modified asthma predictive index (API). Modified API introduced IgE sensitization to milk, egg, and peanuts, as a secondary criterion to predict asthma occurrence in children at risk. In children with at least one parent with atopy (positive allergen skin tests) or doctor-diagnosed asthma, modified API showed a high predictive value for the development of asthma at 6, 8, and 11 years of age (38).

On the contrary, Brockow et al. (39) did not find any correlation between egg sensitization during the first year of life and the onset of atopic disease at 6 years of age. In a 10-year follow-up cohort of 565 children with maternal allergy, Bekkers et al. (40) detected specific IgE antibodies to egg in 10% of cases at 1 year of age. Serum specific IgE antibodies to egg, but not to cow’s milk, at age 1 were associated with an increased cumulative risk for asthma at 11 years. However, no association was found between egg sensitization and asthma attacks at 11 years of age.

Taken together, these data suggest that in subjects with atopic family history (33, 36, 40) or wheezing (36), early sensitization to egg may be useful to identify children who will develop asthma. However, the correlation of egg sensitization and asthma may vary according to age. Therefore, further researches with adequate follow-up are needed to investigate whether results obtained in childhood reflect the risk in adulthood.

## Asthma Induced by Food Allergy

Acute respiratory manifestations of food allergy are rhinitis, laryngeal edema, bronchospasm, and cough (41, 42). Bronchospasm caused by food allergy is a rare condition that is elicited by ingestion or inhalation of the offending food (42). Bronchospasm may develop either in children suffering from asthma or in non-asthmatic patients as part of an allergic reaction to food. Bronchospasm hardly occur in patients with food allergy who have never previously had bronchospasm. In asthmatic children, the rate of asthma attack provoked by oral food challenge tests (OFCs) has been reported to be 2–9% (41, 43–46) (Table 1). Differences in methods and populations between these studies and small number of investigated subjects do not allow us establishing prevalence of asthma exacerbated by food allergy. A study in a general population of asthmatics who underwent a double-blind OFC is warranted to reach firm conclusions. Asthma exacerbated by food ingestion is more common in asthmatic children with food hypersensitivity (47–51). It has been reported that 23% (51) to 40% (45) of asthmatic children with food allergy had wheezing when ingested the offending food. Foods most commonly responsible of asthma exacerbations are egg, cow’s milk, and peanut, but also nuts, fish, soy, turkey, beans, celery, and shellfish have been sparsely reported (46, 51). Asthma exacerbated by food allergy seems to be more frequent in younger children, in children who have or had atopic eczema, or with higher concentration of total IgE (43). During oral food challenge, it rarely happens that offending foods trigger isolated asthma exacerbation (46). Urticaria or rash generally develops prior to the asthmatic symptoms. Asthma is less frequent than cutaneous and gastrointestinal symptoms, but it often occurs during severe anaphylactic reactions to foods.

**Table 1:** **Prevalence of asthma induced by food allergy in children investigated for food allergy or asthma**.

Reference	*N*	Population^a^	Food-related asthma, *n* (%)	Most common triggers	Investigations
Oehling and Baena Cagnani (44)	294	Asthmatic children	24 (8.5)	Egg, cow’s milk, and fish	SPT and OFC
Onorato et al. (43)	300	Asthmatic children and adults	6 (2)	Egg, wheat, corn, fish, and cow’s milk	SPT and/or sIgE, DBPCFC
Hill et al. (49)	100	Children with food allergy	20 (20)	Cow’s milk	SPT and/or sIgE, OFC
Novembre et al. (41)	140	Asthmatic children	8 (5.7)	Egg, cow’s milk, wheat, fish, and peanut	SPT and/or sIgE, DBPCFC
Bock (48)	410	Asthmatic children	72 (17.6)	Egg, peanut, cow’s milk, nuts, soy, wheat, legume, and turkey	SPT, PFT, and DBPCFC
James et al. (47)	205	Children and adults with food allergy	34 (17)	Egg, cow’s milk, soy, wheat, and fish	SPT, PFT, and DBPCFC
Rance et al. (50)	544	Children with food allergy	47 (8.6)	Egg, peanut, cow’s milk, mustard, and cod	SPT and/or sIgE, LFC, SBPCFC
Yazicioglu et al. (45)	50	Asthmatic children	2 (4)	Egg and cow’s milk	SPT and DBPCFC
Rance and Dutau (51)	163	Asthmatic children with food allergy	23 (9.5)	Peanut, egg, cow’s milk, mustard, codfish, shrimp, kiwi, and nuts	SPT and/or sIgE, PFT, and SBPCFC
Krogulska et al. (46)	362	Asthmatic children	9 (2.5)	Egg, cow’s milk, peanut, wheat, and celery	sIgE and DBPCFC

*^a^Children: <18 years old and adults: >18 years old*.

*SPT, skin prick tests; sIgE, specific IgE level; PFT, pulmonary function tests; OFC, oral food challenge; LFC, labial food challenge; SBPCFC, single-blind, placebo-controlled food challenge; DBPCFC, double-blind, placebo-controlled food challenge*.

There is some support for the view that BHR is augmented in children with food allergy who do not present asthma during OFC (42). In 27% (6/22) of asthmatic children with positive DBPCFC, an increase of BHR measured with methacholine inhalation challenge was recorded in the absence of respiratory symptoms (46). This happened more commonly in children with partially controlled or uncontrolled moderate asthma (42, 46). Along this line, the efficacy of elimination diet in asthmatic children has been analyzed by Yusoff et al. (52) in a single-blind study. They found that children who followed a diet without cow’s milk and egg had a significant improvement of peak expiratory flow values compared with those who followed a free diet. Another report showed that in children with food allergy confirmed by DBPCFC who went on an elimination diet, asthma control improved in 54.5% of cases (46).

In some patients, the ingestion of the offending food is a prerequisite for the development of asthma and anaphylactic symptoms during exertion [food-dependent exercise-induced anaphylaxis (FDEIA)] (53). In FDEIA, food intake or physical exercise alone does not induce symptoms. Anaphylaxis is triggered by physical activities only when the patient ingests a particular food (specific FDEIA) or a meal (non-specific FDEIA) prior to exercise. All type of foods can favor the development of the reaction. Symptoms may be induced by both strenuous and mild physical exercise, and they may not occur every time patient practices physical activity. During anaphylactic reaction, about half of patients present cough, chest tightness, or wheezing. Moreover, reduced percent predicted FEV1 values were recorded also in the absence of respiratory symptoms (54).

Inhaled food molecules can elicit clinical hypersensitivity reactions that may be severe and life threatening (55). The majority of patients present respiratory symptoms: asthma, cough, wheezing, and oculorhinitis. Cutaneous manifestations or anaphylaxis can also occur (56). Reactions to inhaled food allergens generally develop in children with a positive history of IgE-mediated reactions after ingestion of the offending food. Clinical reactions following inhalation of fish or shellfish (57), but not of peanuts (58), were often more severe than those induced by oral intake. Occasionally, foods can cause clinical reactions when inhaled while they are tolerated when ingested. These findings suggest that there is a local production of specific IgE at the respiratory tract (57). Another explanation is that digestion may modify allergenicity of food proteins, and respiratory symptoms may be only induced by inhaled unmodified allergens (59).

Airborne food allergens are both in confined spaces and outdoor (for example, at fish market). They are often widespread when food is cut, cleaned, or cooked, especially if food is boiled or steamed (60). Food processing is not always necessary to produce airborne food particles. In children, any food can cause asthma by inhalation; fish, seafood, legumes, peanuts, nuts, and cow’s milk are more frequently involved. The prevalence of allergic reactions to foods triggered by inhalation of food proteins varies from 1 to 10% (61, 62).

It should be noted that dry powder inhalers may contain lactose whose cow’s milk protein contamination usually does not induce allergic reactions (63). However, caution should be exercised in asthmatic children with severe cow’s milk allergy because chest tightness, decline in FEV1, and blood pressure drop have been described after inhalation from some lots of fluticasone/salmeterol dry powder containing lactose (64). Most of lactose in the powder is swallowed rather than inhaled.

## Management

Food allergy should be investigated in the case of rapid onset, unexpected asthma attacks related to food exposure (56). Anyway, exacerbations can occur not only immediately after food intake but also, rarely, in the late phase, even after 24 h (65). Furthermore, it should be taken into account that additives (66, 67) may trigger bronchospasm, too. It is recommended to perform skin prick tests to the suspected foods and, if necessary, the measurement of serum-specific IgE antibodies (56). False-positive IgE tests to food extracts can be frequent, and they may be the result of cross-reactivity between inhalant allergens and foods. When medical history or IgE tests are not convincing, clinical reaction to foods should be ascertained by OFC (56, 68). When OFC is passed, it should be considered to perform a combination food–exercise challenge test to identify children with FDEIA (69). Patients with specific FDEIA undergo exercise test 1 h after eating each type of suspected foods and those with non-specific FDEIA 1 h after a meal. The food-exertion combined test may be negative when additional cofactors, such as extreme temperatures, are necessary to induce symptoms (70).

The avoidance of the offending food is the cornerstone of treatment. A dietitian should provide an age-appropriate diet (71, 72). Patients and caregivers should be educated to avoid unintentional ingestion. Patients with proven reactions induced by inhalation of food particles should understand that they may be exposed to food proteins not only at home but also at school, restaurant, bar, airplane, fish market, or shops (61). The presence of food proteins in inhaled drugs should be checked before administration (64). Instructions on how to recognize symptoms and on proper use of rescue medications, including short-acting beta2-agonists, anti-cholinergic, corticosteroids, and self-injectable epinephrine, should be given. Self-injectable epinephrine should be prescribed not only to children with anaphylaxis to foods but also to children with food allergy at risk for anaphylaxis because of coexisting unstable or persistent asthma. First dose of epinephrine may fail, so patients should carry two epinephrine autoinjectors (73).

## Conclusion

The relationship between asthma and food allergy has been a subject of study for decades. The body of research provides evidence of their coexistence in childhood although degree and mechanisms are still under investigation. In infants, food sensitization, particularly to egg, predicts the onset of asthma. Asthmatic children with food allergy have an increased risk for asthma morbidity and severe asthma, especially during anaphylaxis. Moreover, they have higher levels of biomarkers of bronchial inflammation. However, it remains to be determined whether food allergy may directly affect asthma control. Patients with allergic reactions to food and asthma are at risk for fatal anaphylaxis caused by food allergy. They must be referred to an allergist and provided with epinephrine autoinjectors. Despite apparent rarity, food-induced asthma is severe and often associated with anaphylactic symptoms. It is, therefore, necessary to make an accurate diagnosis to identify the offending foods in order to avoid exposure and to educate patients on how to treat asthma symptoms and anaphylactic episodes.

## Author Contributions

CC, MG, CG, CM, and CPD substantially contributed to the conception and design of the work, acquisition, analysis, and interpretation of data for the work; drafting the work or revising it critically for important intellectual content; final approval of the version to be published; and agreement to be accountable for all aspects of the work in ensuring that questions related to the accuracy or integrity of any part of the work are appropriately investigated and resolved.

## Conflict of Interest Statement

The authors declare that the research was conducted in the absence of any commercial or financial relationships that could be construed as a potential conflict of interest.
